# Global Prevalence of Non-Polio Enteroviruses Pre- and Post COVID-19 Pandemic

**DOI:** 10.3390/microorganisms13081801

**Published:** 2025-08-01

**Authors:** Marli Vlok, Anna Majer

**Affiliations:** 1 Enterovirus and Enteric Viruses Laboratory, Viral Disease, National Microbiology Laboratory, Public Health Agency of Canada, Winnipeg, MB R3E 3R2, Canada; marli.vlok@phac-aspc.gc.ca; 2 Department of Medical Microbiology and Infectious Diseases, College of Medicine, Faculty of Health Sciences, University of Manitoba, Winnipeg, MB R3E 3R2, Canada

**Keywords:** non-polio enteroviruses (NPEV), global, regional, surveillance, environmental, clinical, COVID-19, genotype, prevalence

## Abstract

Non-polio enteroviruses continue to cause numerous epidemics world-wide that range from mild to severe disease, including acute flaccid paralysis, meningitis, severe respiratory infections and encephalitis. Using publicly available data we present a comprehensive global and regional temporal distribution of non-polio enteroviruses, with a focus on highly prevalent genotypes. We found that regional distribution did vary compared to global prevalence where the top prevalent genotypes included CVA6 and EV-A71 in Asia, EV-D68 in North America and CVA13 in Africa, while E-30 was prevalent in Europe, South America and Oceania. In 2020, the COVID-19 pandemic did interrupt non-polio enterovirus detections globally, and cases rebounded in subsequent years, albeit at lower prevalence and with decreased genotype diversity. Environmental surveillance for non-polio enteroviruses does occur and has been used in some regions as an early-warning system; however, further development is needed to effectively supplement potential gaps in clinical surveillance data. Overall, monitoring for non-polio enteroviruses is critical to identify true incidence, improve understanding of genotype circulation, provide an early warning system for emerging/re-emerging genotypes and allow for better outbreak control.

## 1. Introduction

Enteroviruses (EV) are some of the most common viruses infecting humans world-wide [1,2]. Although generally considered innocuous because they are largely asymptomatic or cause mild disease, enteroviruses can cause a wide range of severe and fatal respiratory, epidermal, gastrological, myocardial and neurological diseases [1,3]. The genus *Enterovirus* contains seven genetically distinct species that infect humans: EV-A to EV-D, which comprise the enterovirus (EV), coxsackievirus (CV) and echovirus (E) genotypes, plus rhinovirus (RV) species A to C. EV-A to EV-D contain 116 distinct serotypes while RV-A to RV-C contain 169 distinct serotypes [2,4]. One of the most widely recognized enteroviruses is poliovirus, the etiological agent of poliomyelitis. This virus has caused debilitating paralysis and death in children for centuries [5] and was one of the most feared viruses in the early 20th century [6]. Poliovirus shares many characteristics with other enteroviruses and is a great example of how these viruses can cause deadly regional and global epidemics. With vaccine development and the establishment of the Global Polio Eradication Initiative (GPEI) in 1988, aggressive global vaccination campaigns led to the systematic elimination of polioviruses world-wide, and the virus currently remains endemic in only two countries [7,8]. As poliovirus cases drastically decreased globally, other non-polio enteroviruses (NPEVs) began to emerge and cause severe disease [9]. Overall, enteroviruses have high mutation rates [10], can readily recombine between genotypes and across species [11,12] and are known to be one of the most rapidly evolving viral groups [13]. These features underscore the need for improved global surveillance to better detect non-polio enteroviruses capable of causing severe disease.

Global non-polio enterovirus surveillance activities vary from voluntary to sentinel-based and reportable. Canada and many other countries around the world lack a formal non-polio enterovirus surveillance program. Samples with suspected NPEV etiologies are voluntarily submitted for NPEV genotyping at appropriate laboratories based on symptomology. Similarly, the National Enterovirus Surveillance System (NESS) in the USA is a voluntary passive surveillance system that monitors enteroviruses and parechoviruses [14]. However, not all enterovirus detections are reported to the NESS and genotyping is not routinely performed as part of clinical care. A more expansive sentinel-based surveillance system to monitor NPEV genotypes is needed, especially one that reliably captures outbreaks and severe cases. An enhanced system to capture syndromic NPEV presentations has been implemented in South Korea since 2006 [15,16]. Similarly, the European Non-Polio Enterovirus Network (ENPEN) was formed in 2022, spanning more than 17 European countries to establish standardized surveillance capabilities for non-polio enteroviruses that are associated with hand, foot and mouth disease as well as respiratory and neurological infections [17].

To help fill the gaps in clinical surveillance activities, population level NPEV surveillance through wastewater monitoring is a proposed alternative. Wastewater surveillance has been a valuable tool in monitoring the spread of other viral infections such as poliovirus [18,19,20,21] and recently SARS-CoV-2 [22,23,24]. As the world nears global poliovirus eradication, NPEV wastewater surveillance is recommended by the WHO for polio-free countries to help uncover potential silent transmissions of poliovirus within communities and to better track NPEV circulation [25,26,27]. In fact, a multimodal surveillance system in Colorado, USA, consisting of syndromic, clinical laboratory and environmental surveillance programs was essential to detect and implement actionable public health responses in real-time that helped minimize the regional EV-D68 outbreak in 2022 [28]. Collectively, a robust and reliable NPEV surveillance system will help delineate the true disease burden of NPEVs, improve our understanding of NPEV circulation, provide an early warning for emerging genotypes and allow for better outbreak control measures [1,29,30].

In this study, we were interested in determining the global and regional prevalence of NPEVs using publicly available sequence data prior to and post the COVID-19 pandemic. Global data revealed five genotypes that accounted for more than 60% of the data: CVA6 (18%), EV-A71 (18%), CVA16 (10%), EV-D68 (9%) and E-30 (7%). Regional genotype patterns, however, did vary: CVA6 and EV-A71 were highly prevalent in Asia, with E-30 in Europe, South America and Oceania, while EV-D68 was dominant in North America, and CVA13 was highly prevalent in Africa. The COVID-19 pandemic restrictions did interrupt NPEV incidence globally, diminishing the overall number of unique NPEV genotypes detected post 2020. However, delays in data submissions complicate the post COVID-19 dataset. Although environmental surveillance activities for NPEVs do exist, results are inconsistent between clinical data in many regions, underscoring the need for improvements for effective implementation. Collectively, the data presented here highlight the continued circulation of NPEVs that cause yearly outbreaks world-wide and points to the need for robust NPEV surveillance to effectively monitor genotypes and emerging lineages of public health concern.

## 2. Methods

### Data Source and Analysis

All human enterovirus data were downloaded from the BV-BRC v3.52.11 database [31] on 7 September 2024. Data included in this study consisted of EV positive samples collected between 1977 and 2024. Poliovirus and rhinovirus entries were excluded from the final dataset. As such, non-polio enterovirus (NPEV) in this study refers to all enterovirus entries that are not poliovirus or rhinovirus. The dataset was further trimmed by removing all entries that lacked annotations in the “Geographical Group” field, all entries with either “uncultured enterovirus” in the “Genome Name” field or “*Enterovirus* sp.” in the “Other name” field. Entries that contained “ATCC” in the “Isolation source” field were also removed. The dataset was further subdivided into clinical and environmental specimens based on the “Isolation source” field. Specifically, entries containing derivatives of “sewage”, “wastewater”, “environmental”, “air sample”, or a specific city or town were included as environmental specimens while the remaining were considered clinical specimens.

To determine the most frequently detected global genotypes, the data was further trimmed to consider NPEVs that comprised at least 1% of all detections in any given year over this time frame. All graphs and statistical data were generated using either Microsoft Excel 2016 or GraphPad Prism 10.4.1. Standard deviation (*n* = 3–6) was calculated for the number of unique NPEVs detected prior to and post 2020.

## 3. Results

### 3.1. Global Incidence of NPEVs Detected from Clinical Surveillance Activities for the Past 47 Years

**Figure 1:**
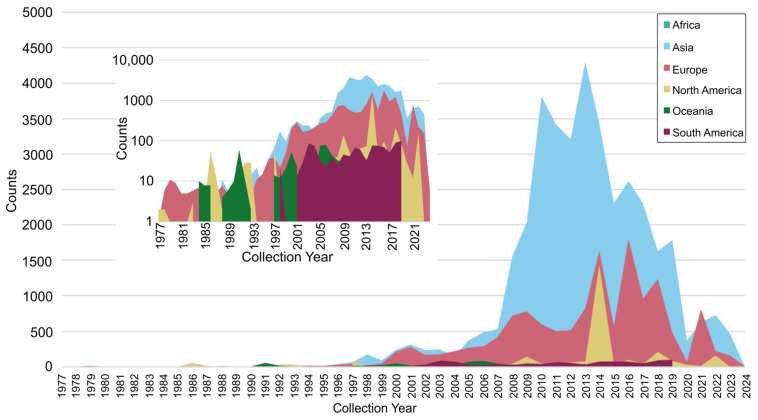
Global counts of NPEV sequences from clinical surveillance data. Data was obtained from the BV-BRC database for samples collected between 1977 and 2024. Inset is the log transformed data.

The BV-BRC database was used to curate publicly available global non-polio enterovirus (NPEV) sequence data that represented samples collected between 1977 and 2024. A total of 59,602 NPEV entries were obtained, spanning data across six different continents. Investigating the data source revealed that Asia was the largest contributor, comprising ~63% (37,686) of all entries in the database. Europe was the next largest contributor, accounting for 23.8% (14,204) of all entries, followed by North America at 5% (2965), Africa at 4.8% (2858), South America at 1.9% (1107) and Oceania at 1.3% (782) (Supplemental Figure S1). Evaluating global NPEV trends over this time period revealed sparse reported case counts (<500) until 2000, when cases began to steadily climb, reaching >3000 cases globally by 2009 (Figure 1). Between 2009 and 2018 inclusive, global case counts surpassed 3000 cases per year, with the highest counts (~6700) occurring in 2014. Not surprisingly, we found a significant drop in EV cases in 2020, which correlated with the COVID-19 pandemic. In 2021 and 2022, there was a clear, albeit modest, upswing in EV detections. Of note, NPEV detections in 2023 and 2024 are a vast under-representation of true cases due to the delay in data submission to publicly available databases (Supplemental Figure S2).

**Figure 2:**
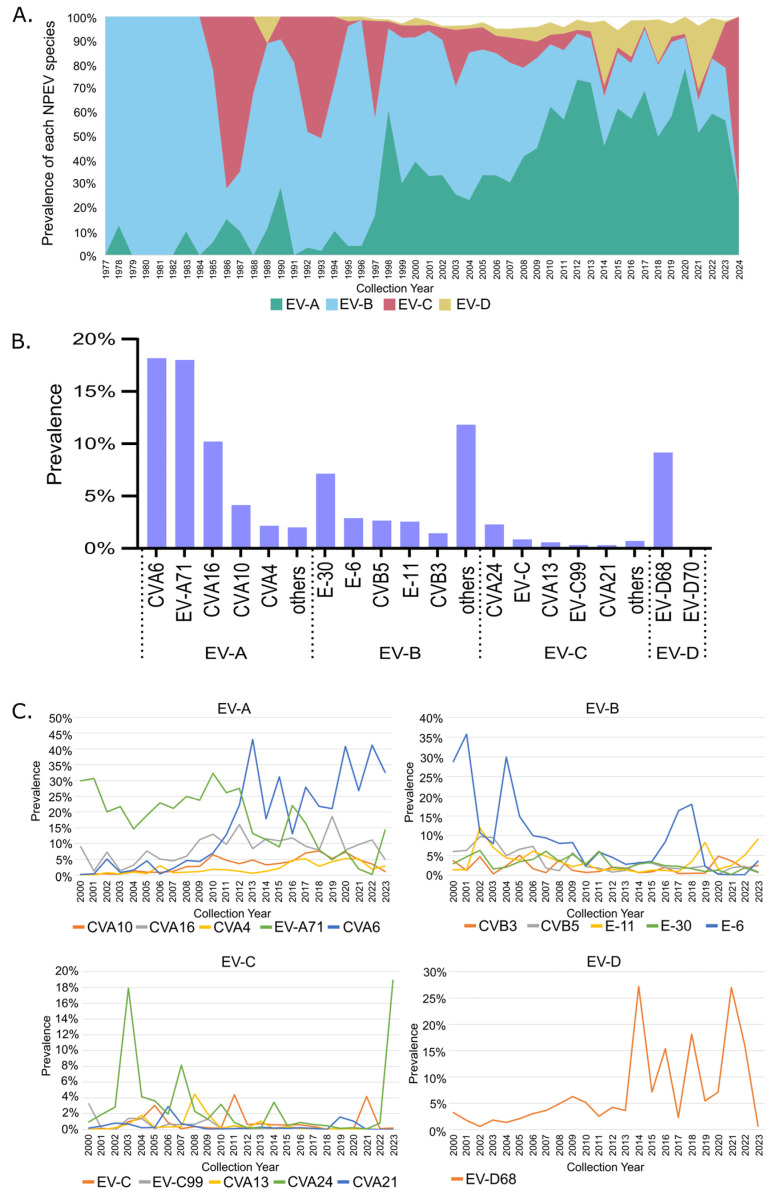
Global NPEV prevalence and the top dominant genotypes. (**A**) Yearly prevalence of NPEV species. (**B**) Top 5 genotypes detected globally across each NPEV species. (**C**) Temporal profiles of the top 5 genotypes detected between 2000 and 2024. Any untyped viruses are depicted as their species.

We were next interested in determining which NPEV species were predominant in the global dataset. The global proportion of each unique NPEV identified over the past 47 years was calculated. The data was further trimmed to consider NPEVs that comprised at least 1% of all detections in any given year over this time frame. This helped showcase the NPEVs that contributed to the global detections as well as pinpoint NPEVs that were repeatedly responsible for outbreaks and continued to circulate within the global population. We found a total of 54 unique NPEV genotypes spanning all four EV species that passed our selection criteria and collectively represented 97% of the global data (Supplemental Figure S3). Temporal trends of the four EV species revealed EV-B predominated prior to 2008 and progressively diminished, while the EV-A species expanded and began to dominate from 2009 onwards (Figure 2A). Although the global prevalence of all non-poliovirus EV-C species remained low, we noted 5 distinct years when detections spiked (1986, 1987, 1992, 1993 and 1997). EV-D was detected in minute proportions prior to the observed expansion in 2014, and although a minor contributor to global prevalence for subsequent years, it continued to persist during and post the COVID-19 pandemic (Figure 2A). Amongst each EV species, only a handful of distinct genotypes were predominantly detected in the global dataset. For EV-A, CVA6 (~18%) and EV-A71 (18%) were the most commonly detected, followed by CVA16 (10%) and CVA10 (4%). For EV-B, the dominant genotype was E-30 (7%); for EV-C, it was CV-A24 (2.3%) and for EV-D, it was EV-D68 (9%) (Figure 2B).

To delineate temporal trends of the dominant genotypes found globally, we further focused on data collected between 2000 and 2023 because global NPEV detections represented >500 cases every year, with 2020 being the notable exception. We found clear temporal shifts and peaks in genotype dominance for all NPEV species. For the EV-A species, EV-A71 was dominant until 2012, at which point CVA6 detections spiked in 2013 (43%) and continued to dominate, albeit at similar or reduced levels. CVA16 peaked in 2019 (18.6%) and largely remained detected at ~10% prevalence since 2009. Both CVA10 and CVA4 remained at low proportions, reaching and maintaining a 5% prevalence since 2010 or 2016, respectively (Figure 2C). For the EV-B species, E-30 was the dominant genotype, surpassing 30% prevalence in 2001 and 2004, followed by a low activity period, and then another resurgence in 2017–2018 when detections surpassed 15% prevalence. The remaining genotypes largely remained detected below 5% prevalence, although E-11 did show a slight upswing in 2019 and 2023. For the EV-C species, detections of CVA24 reached 18% prevalence in 2003 and again in 2023, with smaller peaks of activity observed every 3–4 years, specifically in 2007 (8%), 2010 and 2014 (~3%). The other EV-C genotypes were less prevalent with CVA13 detections surpassing 4% prevalence in 2008 and spikes in EV-C species unable to be genotyped in 2011 and 2021, reaching ~4% prevalence. For the EV-D species, the only notable genotype was EV-D68, and peaks of prevalence can be observed starting from 2014, surpassing 25% prevalence, followed by spikes in detections in 2016 (15%) and 2018 (>15%) and then a notable post pandemic resurgence in 2021 (>25%) (Figure 2C).

### 3.2. Regional Incidence of NPEVs Detected from Clinical Surveillance Activities for the Past 24 Years

**Figure 3:**
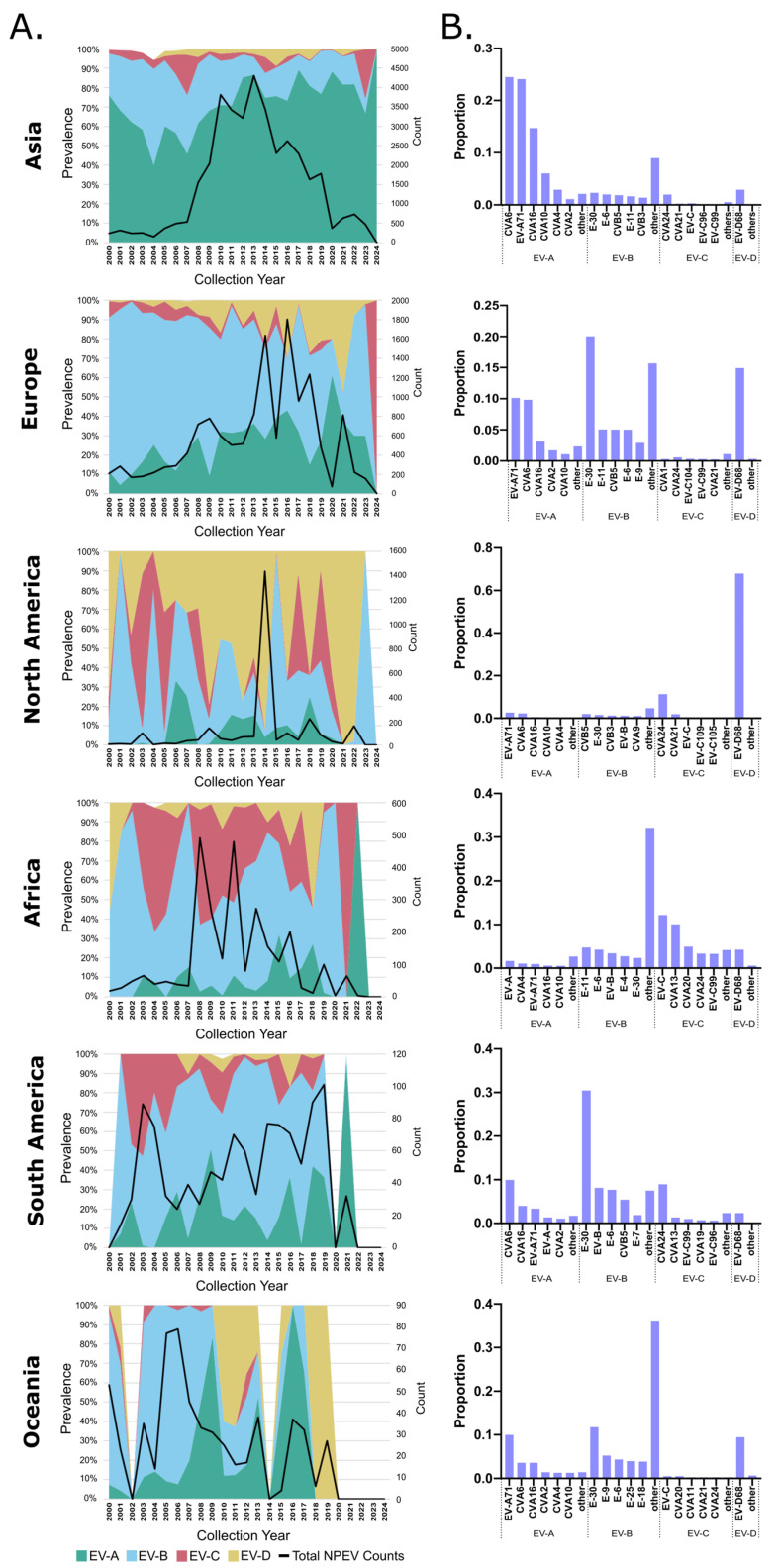
Regional NPEV temporal prevalence and the top 5 dominant genotypes for each EV species. (**A**) Yearly prevalence of NPEV species across each specified geographical region between 2000 and 2024. (**B**) The top 5 dominant genotypes detected for each NPEV species for each geographical location. Any untyped viruses are depicted as their species.

To determine whether the global NPEV trends reflected the regional trends, we subdivided the data into six geographical groups delineated within the dataset (North America, South America, Africa, Asia, Europe and Oceania). This dataset represented collections between 2000 and 2024 because it largely consisted of more than >500 entries per year. We found clear regional-specific trends over time (Figure 3A). Notably, EV-A remained the dominant NPEV species in Asia, accounting for more than 75% of all detections from 2012 onwards. In turn, the EV-B species was dominant in Europe and South America, with notable spikes of EV-A in select years. In North America, the EV-D species was by far the dominant NPEV species detected. In Africa, both the EV-B and EV-C species contributed to the largest prevalence, while a mixture of the EV-A, EV-B and EV-D species was seen in Oceania.

To uncover the potential regional-specific circulation of genotypes, we identified the top five genotypes representing each NPEV species detected in each geographical region. Not surprisingly, certain genotypes were dominantly detected across the globe, while others appeared to have more regional dominance. In particular, EV-A71 and CVA16 were found to be the two dominant EV-A genotypes detected in all geographical regions. Similarly, CVA6 was the top prevalent genotype in most of the world except Africa. Additionally, CVA2, CVA4 and CVA10 were detected in at least four of the six geographical regions, highlighting potential region-specific circulation (Figure 3B). For the EV-B species, E-30 was found to be the most dominant genotype globally detected; the highest levels were found in Asia, Europe, South America and Oceania. E-30 was the second highest EV-B genotype in North America and fifth highest in Africa. Other genotypes within this species, however, were only detected within the top five dominant genotypes in, at most, four out of the six geographical locations (i.e., CVB5 and E-6). For the EV-C species, only CVA24 was found within the top five genotypes globally, while others were found in four out of six geographical locations (i.e., E-C99, CVA21 and the untyped EV-C). For the EV-D species, the only genotype persistently detected globally was EV-D68 (Figure 3B).

**Figure 4:**
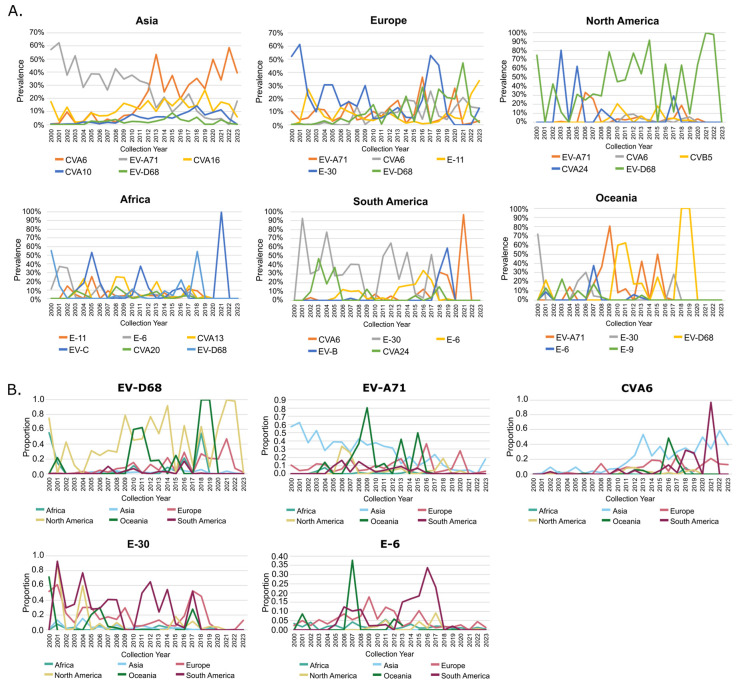
Temporal prevalence of the dominant NPEV genotypes detected in each geographical region between 2000 and 2023. (**A**) Temporal prevalence of the top 5 genotypes identified in each geographical region. (**B**) Temporal patterns of the most commonly detected EV genotypes world-wide where EV-D68 was detected within the top 5 dominant genotypes for 5/6 regions and EV-A71 as well as CVA6 were detected in 4/6 geographical regions, while E-30 and E-6 were detected in 3/6 geographical regions. Any untyped viruses are depicted as their species.

To investigate the temporal shifts and highlight regional-specific peaks of NPEVs that likely corresponded to regional outbreaks, we evaluated the top five dominant NPEV genotypes detected between 2000 and 2023 for each region. In Asia, we noted a progressive temporal switch from the dominant EV-A71 to CVA6 in 2012–2013. Although other NPEV genotypes were responsible for certain peaks in activity, most were found to maintain a prevalence below 20% over this time period (Figure 4). Conversely, E-30 predominated all other genotypes in Europe, surpassing 50% prevalence in 2001 and then again in 2017. EV-D68, although generally not readily detected, did surpass 20% prevalence in several successive years (i.e., 2014, 2016, 2018 and 2021). In North America, EV-D68 detections comprised the largest proportion of data across this time period, with clear spikes in detection across many years. In Africa, the most dominant recently detected genotype was EV-D68, spiking in 2016 and 2018. However, the EV-C species that were not resolved into genotypes contributed to a large proportion of data from this region. In South America, E-30 predominated detections up to 2017. Subsequently, CVA6 detections peaked in 2018, 2019 and 2021, while moderate prevalence (up to 30%) was observed for E-6 from 2013–2017. Similarly to Africa, the untyped EV-B species comprised a large proportion of data between 2018 and 2019. In Oceania, EV-A71 and EV-D68 predominated from 2008 onwards with a notable spike of E-30 in 2017.

Collectively, regional data did highlight the global spread of five highly prevalent genotypes that were detected in at least three of the geographical regions. We noted EV-D68, EV-A71, CVA6, E-30 and E-6 to pass this criterion. Temporal distribution of these genotypes revealed either overlapping peaks of activities or years when peaks of activities diverged across regions (Figure 4B). Specifically, EV-D68 was largely detected globally with peaks occurring largely in North America and Oceania in 2009–2010 but also observed in the rest of the world by 2016. For EV-71, we see a progressive decrease in detections in Asia, while there was an emergence of peaks starting from 2013 in Oceania, Europe and North America. Conversely, CVA6 activity largely commenced in Asia in 2012 and as cases progressively rose in the region, peaks of activity were observed in Europe, Oceania and South America in subsequent years. For E-30, the last major spike in detections occurred in Europe, South America, Oceania and North America in 2017 and remained very low in activity after 2018. Lastly, E-6 temporal detections highlight its persistent presence in Oceania, South American and Europe, although also noted in North America in 2017.

### 3.3. Observed Decrease in NPEV Genotype Diversity Detected Post COVID-19 Pandemic

**Figure 5:**
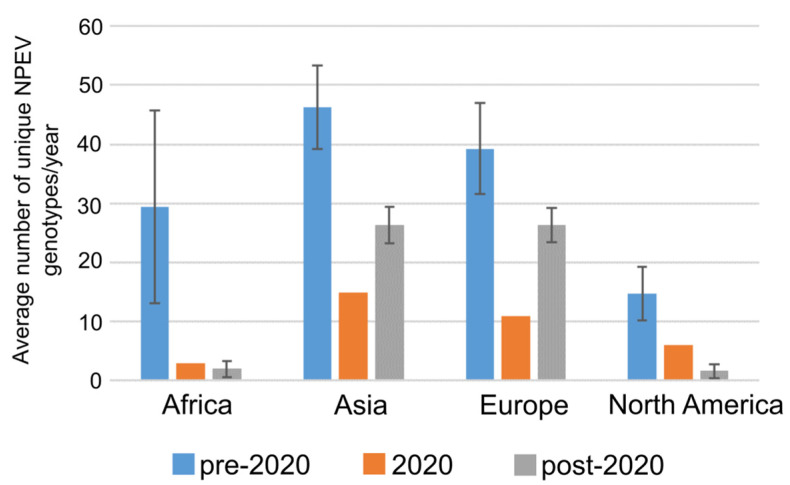
Genotype complexity of NPEVs were decreased as a result of the COVID-19 pandemic. Regional decrease in the number of unique genotypes detected during (2020) and post COVID-19 pandemic (post-2020). Graph represents average number of unique NPEVs detected per year for pre-2020 (2013–2019), 2020 and post 2020 (2021–2023). Standard deviation of the mean is depicted for pre-2020 and post-2020 datasets.

To determine whether NPEV genotype diversity decreased as a result of public health measures imposed during the COVID-19 pandemic, we specifically focused on data from four regions (Asia, Europe, North America and Africa) because they contained consistent information for the years 2013–2023. We chose this extended data range to capture as many unique genotypes based on the fact that: (1) NPEVs have a cyclical pattern of circulation that can span several years of no incidence of detection (Figure 2 and Figure 4) and (2) delays in bulk data submission to public databases can take several years (Supplemental Figure S2). Overall, we found a significant decrease in the number of unique NPEVs detected in 2020 and although marginal increases were observed in Asia and Europe, levels remained significantly below the pre-2020 years (2013–2019 inclusive). Approximately a 30–40% decrease in NPEV genotype diversity was observed in Asia and Europe post 2020, while a staggering decrease of approximately 85–95% of genotypes was noted in North America and Africa (Figure 5). We acknowledge that there could be a delay in data submission to public repositories that could affect regional data since an overall delay of ~1–4 years post detection can be seen in this global dataset (Supplemental Figure S2).

### 3.4. Global Environmental Surveillance of NPEVs

**Figure 6:**
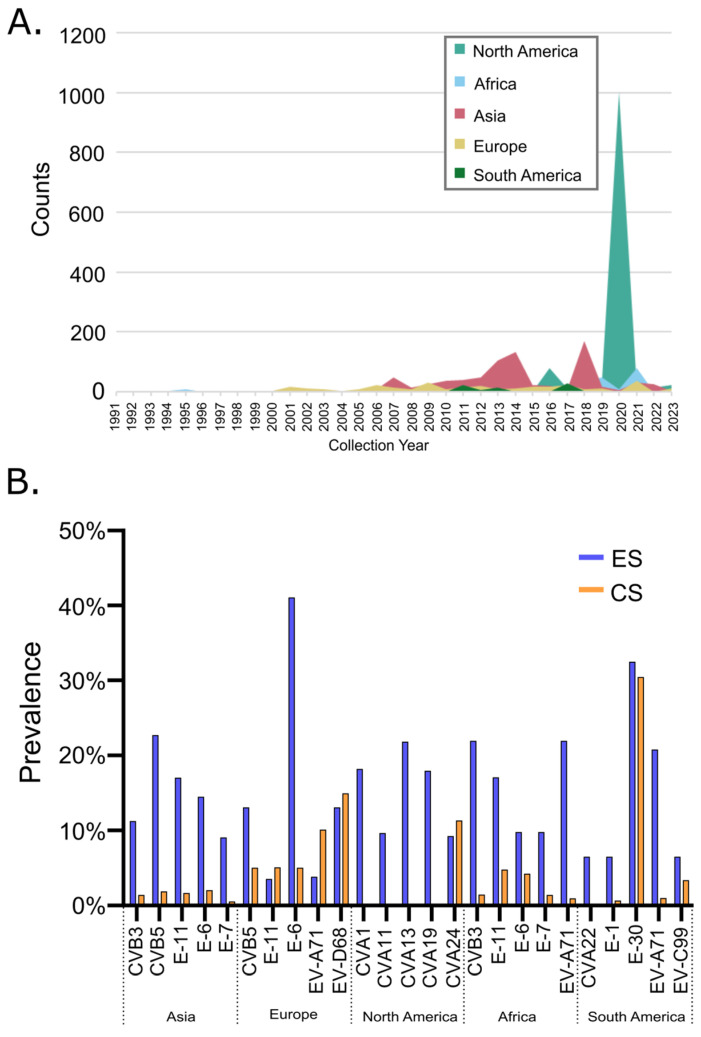
Global environmental surveillance data for NPEV detections. (**A**) Case count data for the 6 geographical regions. Data was obtained from the BV-BRC database. (**B**) The top 5 NPEV genotypes detected between 2000 and 2023 in each geographical region compared to prevalence values of these genotypes obtained from clinical data. ES = environmental surveillance, CS = clinical surveillance.

Publicly available data pertaining to environmental surveillance (ES) is relatively sparse globally (Figure 6A), accounting for a total of 2589 sequences and amassing generally less than 100 NPEV counts per year. A clear spike in NPEV environmental surveillance activities can be observed in North America in 2020, amounting to ~1000 NPEV entries. Although sampling for certain regions appears to be more consistent (spanning >5 successive years), we did not evaluate this data to the granularity of specific countries or cities to determine if the same sites were being sampled over multiple years due to the overall sparsity of the data. To determine whether the total environmental surveillance data aligned with the detections observed through clinical surveillance, we identified the top five dominant NPEVs that were detected between 2000 and 2023 for each geographical region in order to determine whether the same genotypes were identified within the clinical dataset. We found that in select geographical regions, the environmental surveillance data did somewhat trend with the clinical surveillance data. Collectively, three of the top NPEVs detected through clinical surveillance (E-30, EV-D68 and EV-A71) were also one of the top five detected through environmental sampling in some of the geographical regions (Figure 6B). In Europe, EV-D68 was detected at a comparable prevalence of ~15% by both clinical and environmental surveillance activities. Similarly, in South America, E-30 was detected at similar prevalence of ~30% while in North America CVA24 was detected at ~10% in both clinical and environmental surveillance sources. However, a large proportion of data collected by environmental surveillance for Asian, North American and African regions do not represent clinical data.

## 4. Discussion

In this study we present both a global and regional temporal distribution of NPEVs using publicly available data, with a focus on highly prevalent genotypes. Global prevalence indicated that the EV-B species diminished from 2010 onwards while the EV-A species became progressively dominant. However, the global data does represent a large regional bias towards data from Asia and Europe, which accounted for ~87% of all entries. Regional distribution showed a different NPEV profile. Specifically, the EV-A species were highly prevalent in Asia while the EV-B species were highly prevalent in Europe and South America. The EV-D species were primarily detected in North America while in Africa, both the EV-B and EV-C species were prevalent. The Oceania region contained a mixture of the EV-A, EV-B and EV-C species. Our observations largely corroborate findings by others [32,33,34], although some discrepancies were noted. Specifically, we and others [33,34] found that the EV-A species were dominant in Asia, while a recent review found it to be EV-B [32]. This apparent difference is likely a result of including all sequence data in the work presented here while disease-specific entries for HFMD were excluded from the dataset by Brouwer and colleagues [32].

The high prevalence of the EV-A species in Asia largely reflects the disease-specific surveillance activities of the region. In particular, cases of hand, foot, and mouth disease (HFMD) were made reportable by China in 2008 [35], which resulted in a stark increase in EV detections. This undoubtedly contributed to the dominant detection of genotypes such as CVA6, EV-A71 and CVA16 that we saw together corresponded to nearly half (~17,000) of the total detections in the region. This is not surprising because outbreaks of HFMD were readily reported in Asia over the past decades, of which most were attributed to EV-A71, CVA6 and EVA16 [35,36,37,38]. In fact, between 2008 and 2012, there were more than seven million reported cases of HFMD caused predominantly by EV-A71 and CVA16 in China [39]. Interestingly, we noted a temporal shift between two EV genotypes, where EV-A71 was largely prevalent prior to 2012, while CVA6 became prevalent after 2013. The introduction of the EV-A71 vaccines in China likely contributed to this observed shift in circulating genotypes as the vaccination efforts effectively decreased EV-A71 detections over the years but allowed other genotypes such as CVA16 cases to rise in the population instead [38]. The study by Hong and colleagues [38] noted that the vaccines were effective at protecting the population from and decreasing disease severity caused by EV-A71; however, the risk of severe disease increased if infected by other EVs such as CVA16. We found that CVA16 levels largely surpassed 10% prevalence in Asia during 2009 and continued to contribute to outbreaks every 2–3 years, similar to what other reports indicated [37]. As these viruses co-circulate in the population, recombination events have been found in EV-A71, CVA16 and CVA6 [40], and some of these recombination events have been proposed to be responsible for large-scale HFMD outbreaks [41] with improved virulence as observed in animal models [42]. Detections of EV-A71 and/or CVA6 were also observed in Europe, North America, South America and Oceania as recently as 2020–2021 in most regions, further supporting global spread of these viruses [36,43]. The widespread global distribution of these NPEV genotypes further supports the need for more active surveillance and also development of a multivalent vaccine to better control HFMD [36].

In Europe, we found the EV-B species to dominate the dataset, of which E-30 was of highest prevalence. Temporal trends highlight several outbreaks where peaks of prevalence reached 30% or surpassed 50%, depending on the year, with cyclical patterns occurring every 3–5 years and lasting 1–2 years as previously noted [44,45]. The latest E-30 outbreak in Europe occurred between 2017 and 2018, which coincided with the global data for that region. The emergence of two distinct clades that circulated in the region at that time [44] was suggested to be the cause of the observed regional epidemic and led to an increase in neurological symptoms [46]. E-30 was also detected in South America, Oceania and North America in 2017, although the overall data for some of these regions is relatively sparse.

In North America, the most prevalent EV genotype was EV-D68. This is largely due to the outbreak of EV-D68 in 2014, which coincided with widespread regional outbreaks of severe respiratory illness and acute flaccid myelitis in the United States, Canada and Europe, affecting more than 2200 individuals world-wide [47]. EV-D68 detections in North America clearly highlight a cyclical pattern of viral activity every 2 years in accordance with known outbreaks and patterns of resurgence [48]. The association of EV-D68 with severe respiratory and neurological disease spurred the need to incorporate enhanced laboratory-based surveillance activities in the region and globally [49,50], resulting in the likely over-representation of EV-D68 detections as compared to other NPEVs. Our data also highlights the global spread of EV-D68 because it was found within the top five prevalent EVs detected world-wide, with peaks of activity occurring in numerous countries, coinciding with known global outbreaks especially in 2016 and 2018 [51,52,53,54,55,56,57,58,59,60,61,62,63]. Since EV-D68 is largely found in respiratory samples, COVID-19 pandemic restrictions have interfered with viral transmission [64]. However, with the easing of restrictions, the prevalence of EV-D68 in North America quickly rebounded, although largely without causing neurological complications or AFM [28,65,66,67]. This was also observed in Europe by ENPEN, where 93% of cases were due to acute respiratory distress and 6.4% presented with neurological complications during the fall–winter season of 2021–2022 [68]. Although some studies initially supported the notion that the neurotropic properties of EV-D68 were clade-specific [69,70], recent work suggest that is not the case [71,72]. Further work into deciphering the neurotropic properties of NPEV genotypes, such as EV-D68, that have the propensity to cause severe disease is highly warranted.

Detections for Africa, South America and Oceania were relatively sparse, making it challenging to extrapolate on regional EV circulation patterns. In Africa, the EV-B and EV-C species made up the bulk of the data as previously noted [73,74], with CVA13, CVA20, E-11, E6 and EV-D68 being the most prevalent genotypes in the region. These detections correlate with reported outbreaks in the region [54,55,75]. Similarly, South America and Oceania had higher prevalences of EV-B and EV-A genotypes including EV-A71, CVA6 and E-30 [76,77,78,79,80,81,82,83]. All of these detections, however, do highlight that global outbreaks/epidemics can overlap in years of incidence. Improving surveillance activities in these regions as well as globally will undoubtedly increase the resolution for the reliable monitoring of NPEVs, especially genotypes and/or lineages that result in severe human disease.

Data presented here further highlight a clear decrease in EV prevalence during the COVID-19 pandemic both on global and regional levels, observations that have been noted by numerous local NPEV surveillance activities world-wide [84,85,86,87,88,89]. It was suggested that the decrease in EV test positivity rate during the COVID-19 pandemic was not a direct result of a decrease in samples tested since in many instances sample numbers increased [85,90] but rather due to social distancing and implementation of personal protective measures, which led to diminished viral transmission [91,92], especially of genotypes spread largely through respiratory routes [64,85]. Upon the lifting of COVID-19 restriction measures, a resurgence of EV detections was observed, albeit at a decreased overall count compared to pre-pandemic levels and with a decreased genotype diversity, similar to what others have found [85,86,90]. However, EVs generally have cyclical peaks of activity that can span periods of low transmission rate for many years. As such, it is highly possible that additional genotypes will reappear in the near future. Furthermore, the delay in data submission could account for an overall under-representation of the true number of circulating NPEV genotypes post the COVID-19 pandemic. Further work is therefore needed to delineate whether genotype abundance decreased due to the COVID-19 pandemic.

Environmental surveillance for NPEV has been proposed as a supplement to clinical surveillance and to serve as a potential tool to help fill gaps in monitoring NPEV circulation [26,93,94,95]. We noted that the NPEV environmental surveillance data overall represented a sparse dataset globally and regionally. Spatial and temporal sample resolution was largely poor, making it challenging to evaluate the utility of this approach for NPEV surveillance. Historically, ES has been readily used for poliovirus surveillance and continues to be used across the globe to monitor for poliovirus in endemic regions [18], monitor for potential cases of importation [96] and/or to detect silent community transmission and thereby, the extent of an outbreak [97,98]. For NPEVs, however, environmental surveillance is largely inconsistent. We therefore performed a congregated analysis, which revealed that ES was able to detect some of the top NPEV genotypes found through clinical surveillance in several geographical regions evaluated, pointing to the potential utility of NPEV environmental surveillance as previously proposed [93,99,100,101] but also highlighting the need for further evaluation. Due to the sparsity of the data, we could not correlate signals from the ES data with the city or country where genotypes were identified from clinical detections. Nevertheless, many ES studies focused on detecting NPEVs do provide information regarding the circulation of NPEVs in a population, which remains unbiased to the clinical surveillance data [102]. In fact, ES of NPEVs as a result of COVID-19 surveillance identified upsurge of NPEV signal prior to a rise in clinical cases in the region [103], supporting use of ES as an early warning system for public health [28]. However, studies that rely on viral culturing versus molecular detection methods do influence the number and genotypes identified, as previously noted [104]. Due to the high genotype complexity of NPEVs, developing reliable methods to detect low abundant and/or emerging sub-genotypes of NPEVs from environmental specimens is essential to better monitor spatial and temporal distributions [93,104]. Furthermore, population level surveillance of NPEVs may not always be informative, especially if the EV genotype causing outbreaks or serious disease is in relatively low numbers and is therefore undetectable through ES [20]. Method standardization is clearly needed to be able to perform analysis of the data from different regions and the choice of sample site (population sampled) must be considered when implementing environmental surveillance in order to maximize the ability to detect low abundant genotypes that can cause severe disease [20,105,106,107,108].

There are several major limitations of this work that need to be considered. Data captured here represent all reported sequence data that is publicly available and therefore cannot inform on the true incidence of circulating NPEVs within any particular region. Furthermore, these data do not represent baseline surveillance activities but rather capture genotypes causing more severe disease. As such, these focus studies for particular genotypes or diseases are likely to be over-representing the sequence data that is publicly available. This considerably skews the sampling bias representing each geographical region as well as globally, making it challenging to rely on the data for trend analysis and forecasting. Furthermore, NPEV specimens that are genotyped represent various regions within the viral genome including 5′ UTR, complete or partial VP1, VP4/VP2 partial capsid region or whole genome sequences [109]. As a result, sequence data deposited within public repositories such as NCBI varies in purpose of sampling as well as sequence data completion. This further causes challenges for informing on the genetic diversity of circulating genotypes. Finally, clear delays in data submission make it impossible to use public data in real-time to assist in outbreak investigations of seasonal and epidemic NPEVs.

## 5. Perspective

Non-polio enteroviruses continue to cause world-wide yearly outbreaks and epidemics that can result in severe disease. Monitoring for these pathogens, however, is largely poor, making it challenging to obtain a better appreciation of the extent of NPEV incidence and disease burden. Although some level of NPEV surveillance activity does exist globally, overall improvements and method standardizations for NPEV surveillance activities are highly warranted to better understand NPEV circulation patterns, monitor the incidence of NPEV infections and to provide an early warning system that can allow for actionable public health measures to contain outbreaks and/or epidemics.

## Data Availability

No new data were created or analyzed in this study. Data sharing is not applicable.

## Supplementary Materials

The following supporting information can be downloaded at: https://www.mdpi.com/article/10.3390/microorganisms13081801/s1, Figure S1: Total number of NPEV entries publicly available for each geographical region; Figure S2: Observed delay of data submission from sample collection to publicly available databases for each respective geographical region; Figure S3: The top 54 genotypes identified in the global NPEV data surpassed 1% threshold of all detections in any given year between 1977–2024.
