# Supplementary material for: Global Prevalence of Non-Polio Enteroviruses Pre- and Post COVID-19 Pandemic
Source: Microorganisms. 2025 Aug 1;13(8):1801. doi: 10.3390/microorganisms13081801 (PMC12388733; doi:10.3390/microorganisms13081801)
Supplement: Supplementary file 1 [file microorganisms-13-01801-s001.zip › microorganisms-3748588-supplementary.pdf]

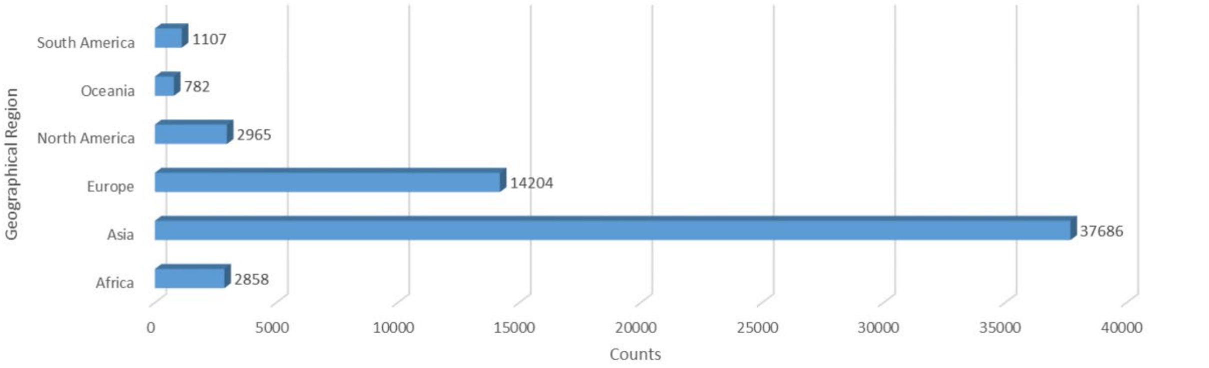

Supplemental Figure S1. Total number of NPEV entries publicly available for each geographical region.

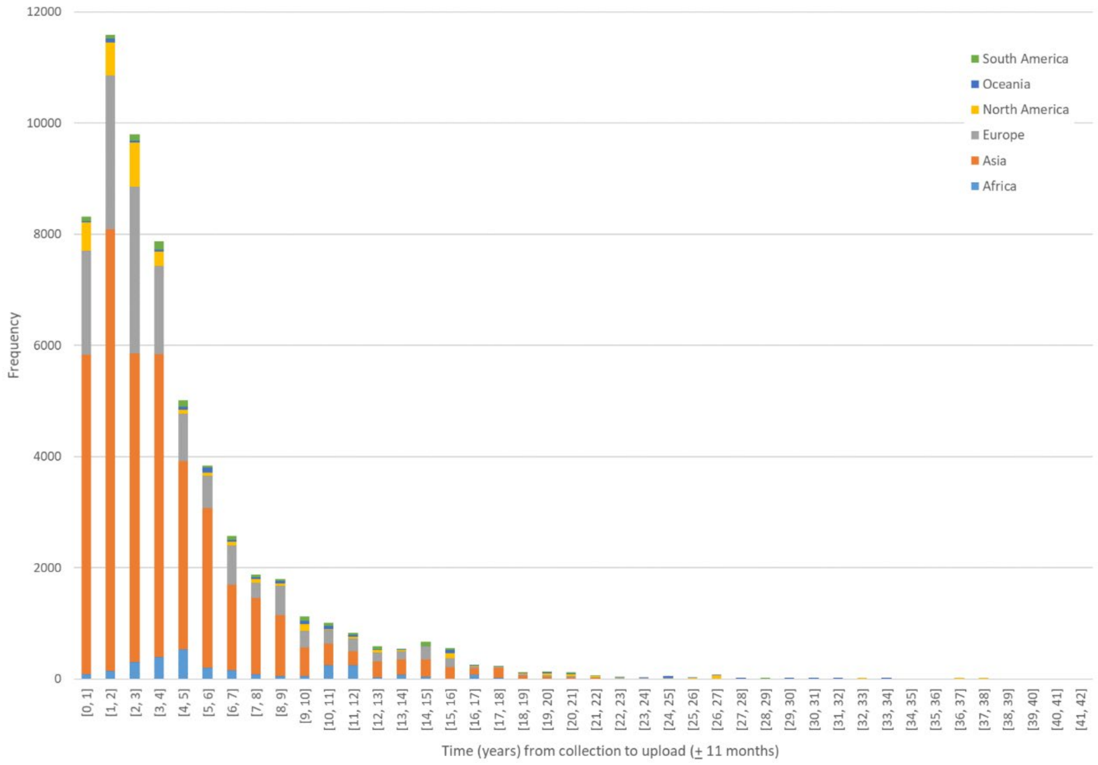

Supplemental Figure S2. Observed delay of data submission from sample collection to publicly available databases for each respective geographical region.

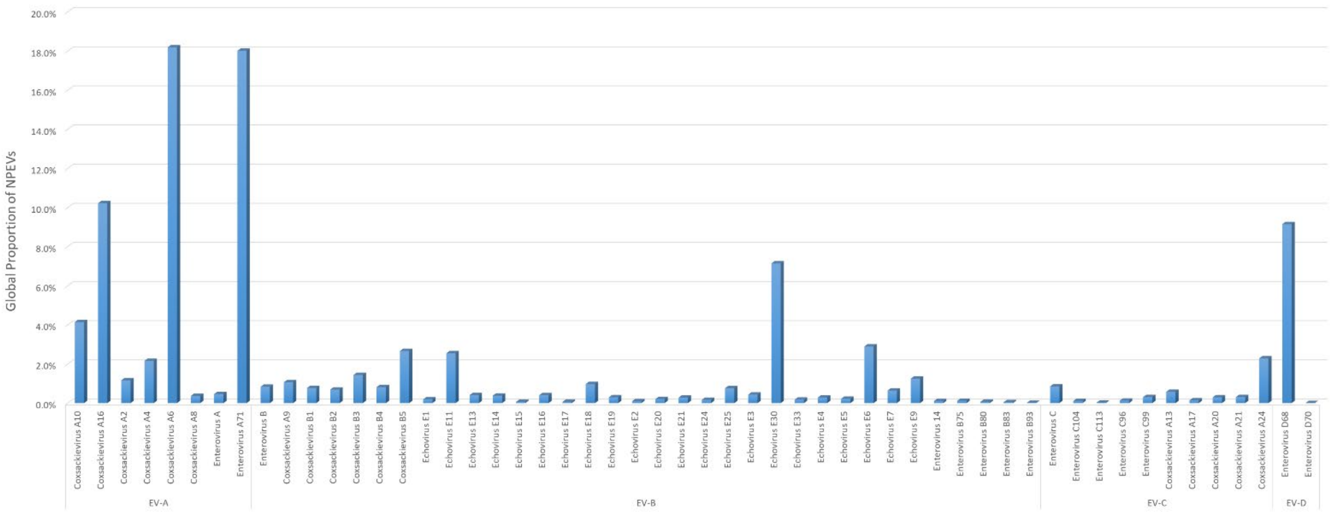

Supplemental Figure S3. The top 54 genotypes identified in the global NPEV data surpassed 1% threshold of all detections in any given year between 1977-2024.
